# Another lesson from beautiful monsters: the case of 'sex reversals' in the Ammonoidea and their significance

**DOI:** 10.1186/s12862-021-01857-y

**Published:** 2021-06-26

**Authors:** Camille Frau, Pierre-Yves Boursicot

**Affiliations:** 1 Groupement D’Intérêt Paléontologique, Science et Exposition, 35 Impasse lieutenant Daumas, 83100 Toulon, France; 2 Present Address: 14, rue Joannes, R49450 Villedieu-la-Blouère, France

**Keywords:** Ammonoidea, Sexual dimorphism, Intersexuality, Pathology, Jurassic

## Abstract

**Background:**

Expression of sexual dimorphism is recognised in various fossil groups of molluscs such as the Ammonoidea, an extinct group of shelled cephalopods. During the Mesozoic, the best-documented sexual dimorphic examples are seen in the Jurassic superfamily Perisphinctoidea. It is usually expressed by distinct adult size and apertural modifications between the antidimorphs. Putative males (otherwise referred to as microconch) are small in size and develop lappets at the end of the shell while the females (macroconch) are larger and bear a simple peristome. Dubious cases are, however, known in that superfamily, which often relate to taxonomic biases or lack of diagnostic characters, and some others expose ontogenetic anomalies illustrated by ‘sex reversals’ in the shell morphology and ornamentation.

**Results:**

The discovery of two specimens of the Callovian Aspidoceratidae *Peltoceras athleta* (Phillips), having both female and male features, questions the significance and causes of ‘sex reversals’ in the Ammonoidea. The two specimens have started with the macroconch ontogeny of *Peltoceras athleta* and show an apparent change toward maleness in the adult, as illustrated by their rounded whorl section, ribs retroversion, fading of the tubercles and lappets typical of the microconchs. Few other cases of female-to-male, as well as male-to-female ‘sex reversal’, are known in the fossil record, all belonging to the Jurassic Perisphinctoidea (families Perisphinctidae or Aspidoceratidae). Since all Jurassic Perisphinctoidea are strictly gonochoristic, these ‘sex reversals’ are pathological in nature and are herein referred to as a new forma-type pathology: namely “forma hermaphrodita”.

**Conclusions:**

In the absence of any clear evidence of injury or parasitism, we hypothesize that such “forma hermaphrodita” individuals illustrate pathologic cases of intersexuality. Little is known about the ammonoid soft parts, and it is not possible to determine which internal sexual organs occur in specimens having both male and female external shell features. Abnormal feminisation and/or masculinisation also occur in modern cephalopods, the latter also grouping only gonochoric species. This phenomenon is similarly illustrated by a change in the adult body size and a mixing of both female and male structures. In that case, intersexuality is either advantageous in the population or caused sterility. The causes of intersexuality are not clearly established but environmental pollutants are evoked in modern cephalopods because they act as endocrine disrupters. ‘Sex reversals’ and/or non-functional reproductive abnormalities have also been caused by endocrine disrupters in various gonochoric gastropods species, but infestation, genetic abnormalities, temperature fluctuations or viruses are multiple causes, which can stimulate or inhibit neural-endocrinal activity by direct gonadal influence, and ultimately lead to feminisation or masculinisation in fishes, isopods, crustaceans, and gastropods as well. Regardless of whether “forma hermaphrodita” is due to an exogenic or endogenic cause, the record of intersex Perisphinctoidea in the Jurassic can be explained by the ready recognition of dimorphic pairs, and the easy collection of large and sufficiently preserved fossil palaeopopulations in which intersex specimens have statistically more chance to be found.

## Background

Dimorphism of sexual nature has been reported in various fossil groups of molluscs by analogy with recent species. De Blainville [1] first suggested that sexual dimorphism occurs in the Ammonoidea—an extinct group of shelled cephalopods—by comparison with the living nautiloid species *Nautilus pompilius* [2]. Nowadays, sexual dimorphism in ammonoids is widely accepted and its palaeobiological criteria are based on (*i*) a change in shell morphology, ornamentation, and aperture, (*ii*) similar early developmental stages, (*iii* & *iv*) same stratigraphic range and overlapping geographic occurrence, (*v*) common ancestors, (*vi*) similar numerical ratio between antidimorphs through time and throughout the evolution of the clade [3].

During the Mesozoic, the Jurassic Perisphinctoidea provide the best-known examples of sexual dimorphism (e.g. [4–17]. In this superfamily, the sexual dimorphism is most often expressed by distinct adult size and apertural modifications. Supposed males (their conchs are referred to as microconchs, [m]) are usually small in size and develop lappets at the end of the growth while the females (macroconchs, [M]) are distinctly larger and bear a simple peristome.

However, many cases of sexual dimorphism remain dubious due to taxonomic biases and lack of diagnostic features in the adult [18]. An increase of doubtful cases is observed in the Perisphinctoidea between the Callovian and the Kimmeridgian stages, as the ones listed by Brochwicz-Lewiński and Różak [19]. These authors have illustrated apparent ‘sex reversals’ in the adult shell of various perisphinctoid species known to be dimorphic. The authors thus concluded that these specimens *“represent a new type of dimorphism not encountered in other groups of ammonites and that […] the hypothesis of the sexual dimorphism is not so universal as it was considered to be*''.

The discovery of two specimens of the Callovian Aspidoceratidae *Peltoceras athleta* [20], having both male and female features, questions the significance of these ‘sex reversals’ in the Ammonoidea.

## Material and methods

### Origin of the material

**Fig. 1:**
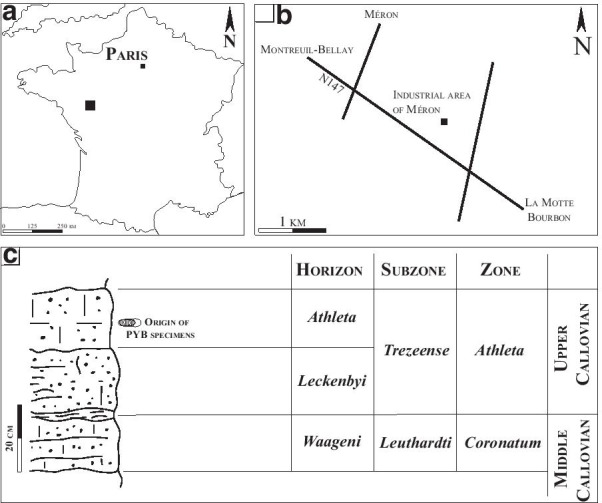
Location of the Méron locality in the vicinity of Montreuil-Bellay (Maine et Loire, France), and its litho-, and biostratigraphic scheme (modified from [21])

The two specimens of *P. athleta*, labelled mbe.9305 and mbe.1401 were collected by one of us (P.-Y.B.) in the industrial area of Méron, near Montreuil-Bellay, Maine-et-Loire, France (Fig. 1A–B). There, Bonnot et al. [21] documented two condensed, oolithic limestone beds of late Callovian age. These beds yield abundant and well-preserved dimorphic-paired ammonite populations (see [21–23]). The studied material originates from the upper limestone bed dated to the *Peltoceras athleta* Horizon, which characterizes the upper *P. athleta* Zone (Fig. 1C).

## Method

**Fig. 2:**
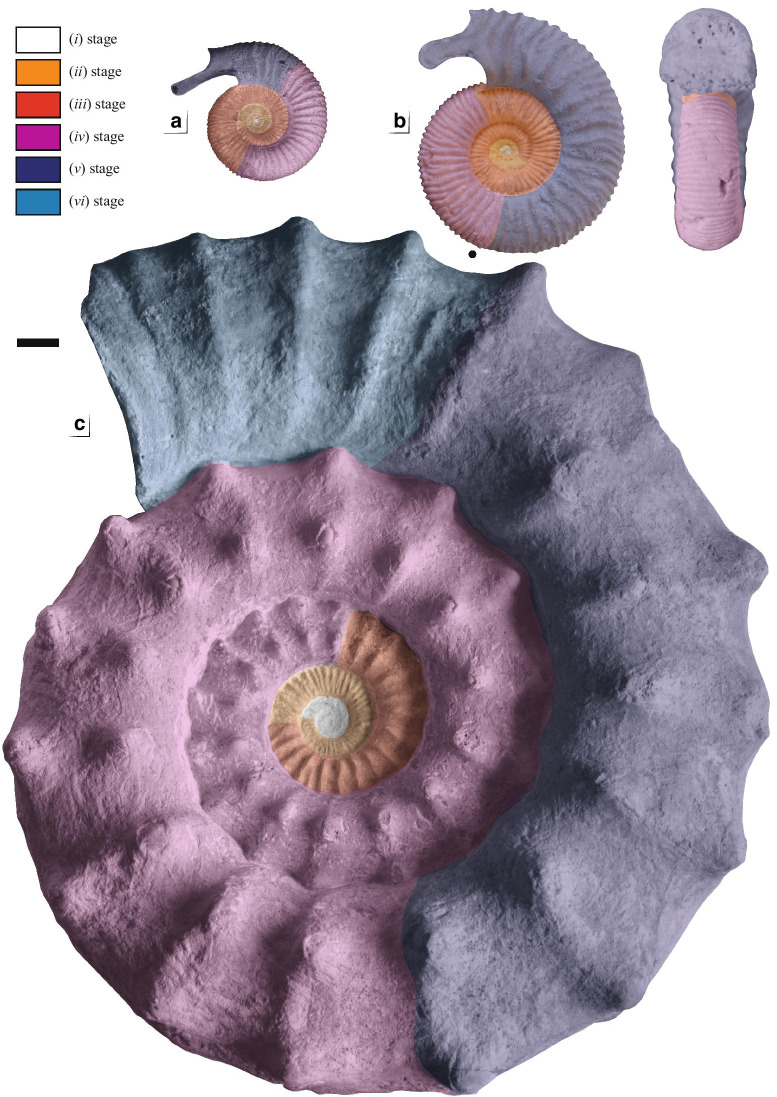
Comparison of selected [m] and [M] morphotypes of *Peltoceras athleta*: **A** *Peltoceras athleta* [M]; **B** *Peltoceras athleta annulosum* [m], **C** *Peltoceras athleta pseudotorosum* [m], and the six (*i* to *vi*) ornamental stages defined by Bonnot [25]. All specimens from the Montreuil-Bellay area (PYB collection). Black dots indicate the end of the phragmocone. Scale bar is 10 mm

Shell parameters and ornamental features of the two specimens have been compared to the ontogeny and intraspecific variability of the dimorphic species *P. athleta* documented by [24, 25]. Two macroconch (e.g. *Athleta* Phillips and *Baylei* Prieser) and two microconch (e.g. *Annulosum* Quenstedt and *Pseudotorosum* Prieser) morphotypes of *P. athleta* are recognized by the same author. Typical representatives of the *Athleta*, *Annulosum* and *Pseudotorosum* morphotypes are herein illustrated for comparison (Fig. 2). (23, p. 267–272) provided a detailed biometric database of shell parameters of the four morphotypes based on juvenile and adult specimens from references localities of France and Switzerland. The database contains standard measurements of the shell, given in millimetres and as percentages of total diameter. The following abbreviations indicate: Dmax = maximum diameter; Uw = umbilical width; Wh = whorl height; Ww = whorl width and K = number of ribs per half a whorl. The ratios of morphological features (Uw/D, Wh/D, Wb/D and Wb/Wh) and rib density (K/D) to diameter are investigated and compared the dimensions of our specimens (Table 1). The coiling terminology follows that of Klug et al. [26].

**Table 1:** Dimensions of the studied *P. athleta* individuals labelled mbe.9305 and mbe.1401 from Méron (PYB collection)

	D	Uw	Wh	Ww	K	Uw/D	Wh/D	Ww/D	Ww/Wh	U/Wh
mbe.9305	66.6	33	19	22	9	0.4954955	0.2852853	0.3243243	1.1368421	1.7368421
mbe.1401	62.3	30	18.9	21	9	0.4879615	0.3033708	0.3306581	1.0899471	1.6084656
Mean value	64.5	32	19	21	9	0.4917285	0.294328	0.3274912	1.1133946	1.6726539

According to (23, p. 272–273), six ornamental stages characterize the ontogeny of *P. athleta* macroconchs (Dmax ~ 240 mm):

(i) Smooth initial whorls with a sub-circular whorl section.
(ii) Approximated, prorsiradiate, bifurcate and single ribs that cross the venter.
(iii) Ribs become radial and spaced.
(iv) Appearance of two lateral tubercles; Whorl section become markedly depressed and trapezoidal.
(v) Disappearance of intercalate ribs on the venter.
(vi) Broad single ribs that cross the venter.

The microconchs (Dmax ~ 110 mm) possess the three first ontogenetic stages of the macroconchs but develop a more circular whorl section. In the microconchs, the fourth stage differs by rectiradiate ribs forming a slight chevron pattern on the venter while the fifth stage develops spaced, strong, rursiradiate, bifurcate ribs approaching an aperture with lappets. The sixth stage is lacking*.* These stages are herein illustrated in both macro-, and microconchs (see Fig. 2).

## Results

### Shell shape

**Fig. 3:**
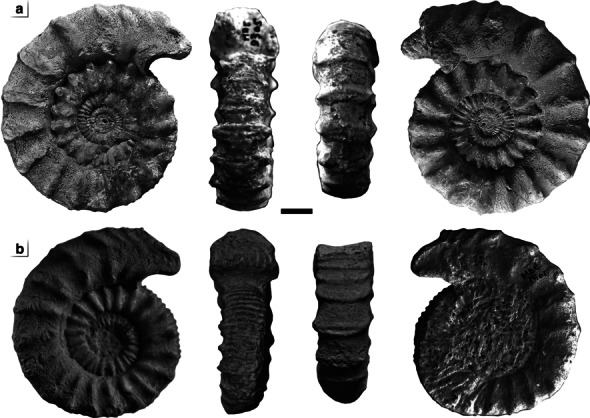
The anomalous individuals of *Peltoceras athleta* from the Upper Callovian (*P. athleta* Zone) of Méron. Specimens with lappets (A: n° mbe.9305; B: n° mbe.1401; PYB collection) showing a typical macroconch ornamentation on the phragmocone, passing to a microconch-like morphology approaching the aperture. White dots indicate the end of the phragmocone. Scale bar is 10 mm

**Fig. 4:**
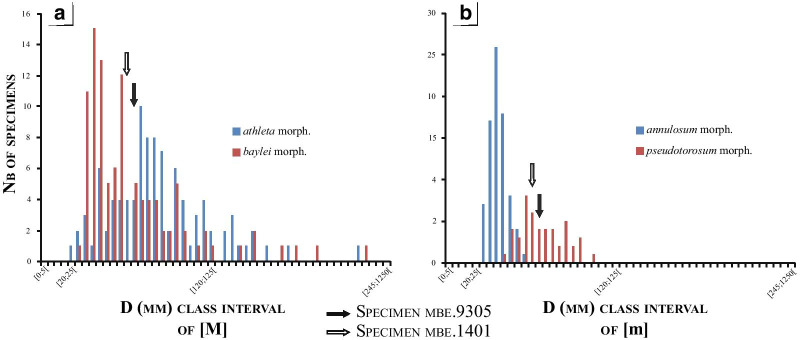
Comparison of specimens mbe.9305 (black arrow), and mbe.1401 (white arrow) with the diameter frequencies of (**A**) *Peltoceras athleta* macroconchs, and (**B**) *Peltoceras athleta* microconchs databased by Bonnot [25]

The two studied specimens are illustrated on Fig. 3A and B, respectively. Both specimens mbe.9305 and mbe.1401 are characterized by a moderate size (62.3 < D < 66.6 mm). They share an extremely discoidal (Ww/D ~ 0.33), weakly depressed (Ww/Wh ~ 1.11), very evolute (U/Wh ~ 1.7) subophiocone coiling (Uw/D ~ 0.49). Specimen mbe.9305 is the most complete individual with almost four preserved whorls. Regarding their adult diameter, the two specimens fall in the peak of the normal distribution of the macroconch diameters (Fig. 4A). They are much larger than the *P. athleta annulosum* microconchs but fall in the normal distribution of the *P. athleta pseudotorosum* diameters (Fig. 4B).

**Fig. 5:**
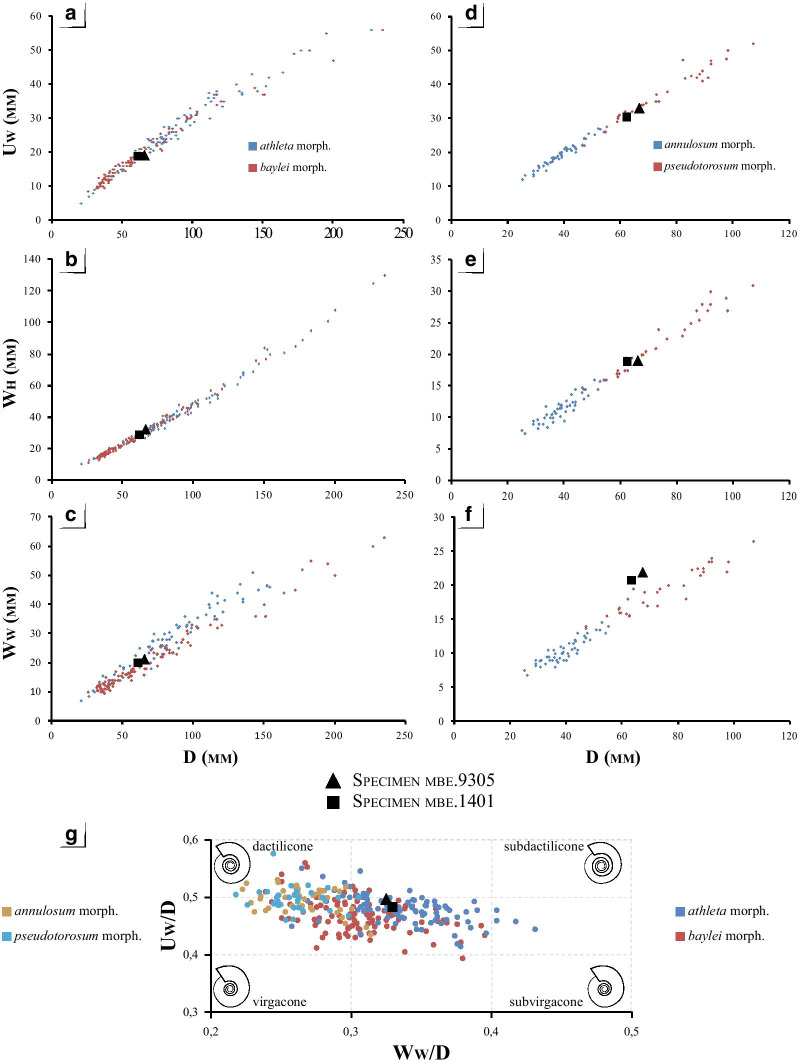
Comparison of mbe.9305 (black triangle), and mbe.1401 (black square) with dimensional shell parameters of *Peltoceras athleta* databased by Bonnot [25]. [M]: Variation of the umbilicus length (**A**); height (**B**), and thickness of the whorl (**C**) with the diameter. [m]: Variation of the umbilicus length (**D**); height (**E**), and thickness of the whorl (**F**) with the diameter. **G** compile the coiling variability of both *Peltoceras athleta* macro-, and microconchs databased by Bonnot [25], and show the position of the studied specimens

Shell parameters of the specimens are compared to the bivariate analysis of the four morphotypes of *P. athleta* provided by Bonnot [21]. The dimensional parameters growth of the shell (Wh, Ww and Uw as function of D—Fig. 5A–F) show homogeneous scatters around the mean curve (with R^2^ still very high ± 0.9) in each case. The growth of those parameters is isometric and harmonic and corresponds to the relationship Y = bD. Regardless of the shell parameters, specimens mbe.9305 and mbe.1401 have greater affinities with the antidimorphs *P. athleta athleta* and *P. athleta pseudotorosum*. Only the whorl width index (Ww/D) of the two specimens deviates from that of the two microconch morphotypes, and better fits into the point cloud of the macroconchs (compare Figs. 5C and F).

Finally, the subophiocone coiling of specimens mbe.9305 and mbe.1401 compares well to that of the macroconchs, since the microconchs have dactilicone to ophiocone conch shapes, rarely subophiocone (Fig. 5G).

### Ornamentation

**Fig. 6:**
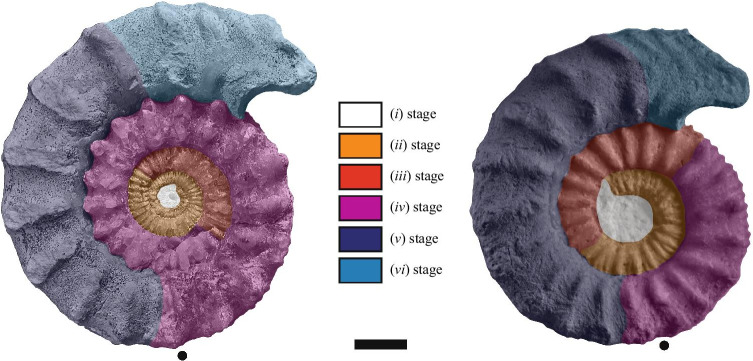
Drawing of the six ornamental stages (*i* to *vi*) of *Peltoceras athleta* as defined by Bonnot [25] on specimens (**A**) mbe.9305, and (**B**): mbe.1401. Black dots indicate the end of the phragmocone. Scale bar is 10 mm

The two sides of specimen mbe.9305 have a similar ornamentation and succession of ontogenetic stages (Fig. 6). This conclusion cannot be reached for specimen mbe.1401 due to the poor preservation of the right flank. No scar or pathological shell compensation is discernible except for the worn inner whorls. Comparison with the ornamental sequence of both macro-, and microconchs of *P. athleta* is as follows:

- The stage (*i*) is lacking in both specimens. Their ornamentation starts directly with the stage (*ii*), which occupies one whorl and a half in specimen mbe.9305. This stage is more anarchic in specimen mbe.1401 as illustrated by simple, bifurcate, polygyrate primary ribs, and irregular intercalatories not typical of that stage.
- The stage (*iii*) is present in the two specimens and occupies half of a whorl. Ribs are more robust and spaced in specimen mbe.1401. They start to develop slight thickenings at the future emplacement of the tubercles.
- The stages (*iv*) and (*v*) are typical of *P. athleta* macroconchs and illustrated by strong bituberculate primary ribs, and the progressive disappearance of intercalatories on the venter. These ontogenetic stages are much longer in specimen mbe.9305 than in the other one since it covers one whorl and a half. First peri-umbilical and upper lateral tubercles of the stage (*iv*) occur at a diameter of 24.3 mm in specimen mbe.9305, and at 35.4 mm in the specimen mbe.1401. The boundary between the phragmocone and the body chamber is located at the upper part of the stage (*iv*) in both specimens. It is at D ~ 43.2 in specimen mbe.1401, and at D ~ 49.4 in specimen mbe.9305. The whorl section is compressed and trapezoidal.
- The stage (*vi*) is modified in both specimens compared to typical macroconchs. It develops approximated, strongly flexuous single or bifurcate ribs crossing the venter, followed by a slightly projected aperture with lappets typical of the microconchs. The section is markedly sub-rounded at the aperture of mbe.1401 and compares well to that of the *P. athleta* microconchs.

**Fig. 7:**
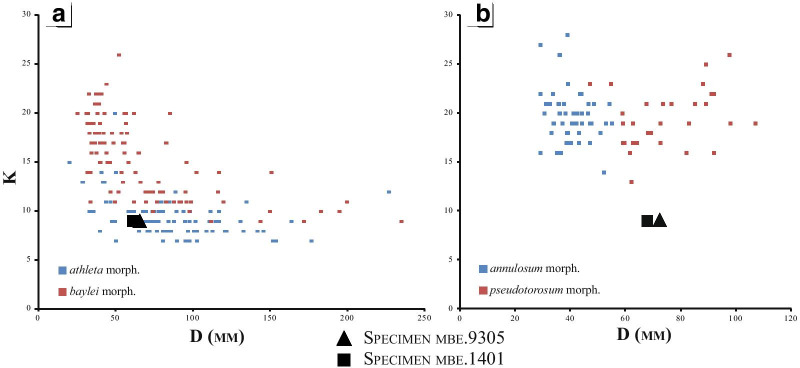
Comparison of the number of primary ribs with the diameter of mbe.9305 (black triangle), and mbe.1401 (black square) (black triangle) databased by Bonnot [25]: (**A**) *Peltoceras athleta* macroconchs [M], and (**B**) *Peltoceras athleta* microconchs [m]

According to the criteria of Bonnot [25], such ornamental sequence conforms well to that of the *Peltoceras athleta baylei* macroconch morphotype by their long-ribbed stage (*ii*), late appearance of stage (*iv*) with reduced peri-umbilical tubercles, and persistence of stage (*v*) in the adult. They, however, develop a sub-rounded whorl section, strong rib retroversion, and a lappet at the end of the shell which are typical features of the *P. athleta pseudotorosum* microconchs. Regarding the number of primary ribs during the ontogenesis (Fig. 7A–B), our specimens fall in the variability of the two macroconch morphotypes. The low density of primary ribs on the body chamber excludes these specimens to the rib variability of the *P. athleta* microconchs.

## Discussion

### 'Sex reversals' in ammonoid shells and definition of a new forma-type pathology

The species *P. athleta* is widely identified as a dimorphic Aspidoceratidae, including large-sized macroconchs with six ornamental stages and a simple aperture *versus* small-sized microconchs with lappets and modified/truncated ornamental sequence [25], and references therein). The individuals at our disposal start with a female ontogeny typical of the *P. athleta* macroconchs and show an apparent change toward maleness in the adult. The ornamental parameters of their phragmocones conform well to those of the *P. athleta baylei* macroconch morphotype, but the adult ornamental modifications better compare to that of the *P. athleta pseudotorosum* microconch morphotype (i.e., low density of primary ribs on the outer whorl, ribs retroversion, and fading of the tubercles). The deviation in the macroconch ornamentation also concerns the whorl section. It is typically depressed and trapezoidal during the bituberculate stage (*iv*) but becomes sub-rounded in the outer whorl; the latter being characteristic of the microconchs. There is no strong deviation regarding the dimensional parameters, except for the conch shape which is much more extremely discoidal than typical microconchs. As such, the expression of a ‘sex reversal’ seemingly affects both the ornamentation and the shell shape during the growth, and the general dimensional parameters to a lesser extent.

**Fig. 8:**
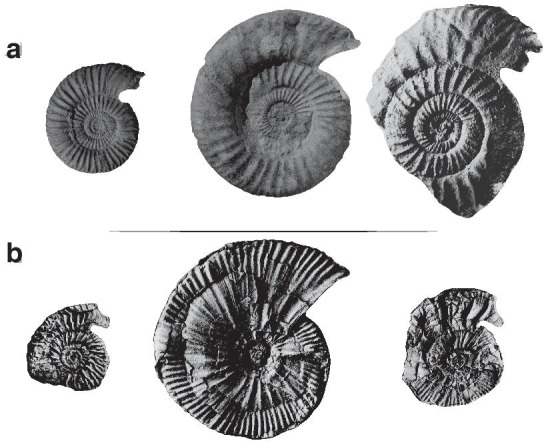
Illustration of female-to-male “sex-reversals” in the Perisphinctoidea: **A** the Kimmeridgian dimorphic Perisphinctidae *Subnebrodites* (pro *Idoceras*) *planula* (Hehl). Typical [m] and [M] are from Schweigert and Kuschel [48] and the “forma hermaphrodita” is from Brochwicz-Lewiński and Różak [19], and doubtfully (**B**) the Oxfordian Perisphinctidae dimorphic pair *Microbiplices* [m]—*Ringsteadia* [M]. All specimens are from Sykes and Callomon [26]. In both cases, the putative “forma hermaphrodita” individuals have the shell features of the [M] but they bear a lappeted peristome typical of the [m]. Specimens are figured using the original scale

A similar ‘sex reversal’ has been reported by Brochwicz-Lewiński and Różak [19] in the Kimmeridgian dimorphic Perisphinctidae *Subnebrodites* (pro *Idoceras*) *planula* (Hehl) (Fig. 8). The figured specimen has the shell features of the macroconchs, but it bears a large lip-like peristome typical of the microconchs at the end of the shell. The significance of the apertural structures has been much debated in the literature, but it is now accepted that they correspond to the ultimate stage of sexual maturation of the males [8, 18]. As such, the abovementioned specimens illustrate female-to-male ‘sex-reversals’.

The phenomenon would also occur in the Oxfordian Perisphinctidae dimorphic pair *Microbiplices* [m]—*Ringsteadia* [M] as suggested by 19, pl. XXXII, Fig. 1), but the figured specimen lacks a clear peristome with lappets for confirmation. Furthermore, the figured specimen rather should be re-assigned to the Kimmeridgian aulacostephanid genus *Vielunia* according to Rogov (pers. comm. 2021), which microconchs belongs to the genus *Prorasenia* [27]. Parent et al. [10] also reported a case of ‘sex reversal’ in *Ringsteadia caledonica*. This would be based on the specimen of (Sykes and Callomon [28], pl. 121, Fig. 9) (Fig. 8B). According to Parent et al. [10], the latter figured specimen develops a macroconch sculpture through its growth but has a microconch peristome at the end of the shell. It seems, however, that this case is not as straightforward as it may seems because the “*microconchs of* [*Ringsteadia caledonica*] *have a similar style of ribbing to the macroconchs, but the adults have lappets*” (Matyja et al. [29], p. 389).

**Fig. 9:**
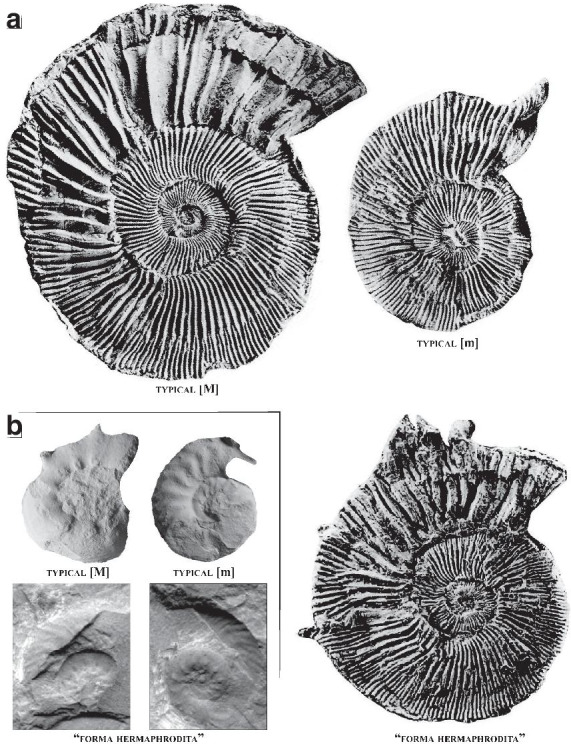
Illustration of male-to-female “sex-reversals” in the Perisphinctoidea: **A** the Kimmeridgian dimorphic Perisphinctidae *Pectinatites* (*Virgatophinctoides*) *reisiformis* Cope. All specimens are from Cope [30], and (**B**) the Kimmeridgian Aspidoceratidae dimorphic pair *Physodoceras* [M]—*Sutneria* [m]. All specimens are from [49]. In (**A**), the “forma hermaphrodita” individual appears intermediate in size between the [m] and [M], has the sculpture typical of the [M], but sporadically develops structures resembling those of the horn of the [m]. In (**B**), the “forma hermaphrodita” individual starts with a bituberculate sculpture typical for female and develops the ornamentation and lappet typical of the [m] on the outer whorl. Specimens are figured using the original scale

It is worth noting that male-to-female ‘sex reversals’ are also reported in the fossil record. For example, (30, p. 16) reported four out of sixty specimens (6%) of the Kimmeridgian Perisphinctidae *Pectinatites* (*Virgatophinctoides*) *reisiformis* Cope, which "*appear to be normal macroconchs but have on their inner whorls structures resembling those of the horn of the microconch*" (Fig. 9A). Also, four *Pectinatites* specimens are "*intermediate in size between the two (micro- and macroconch) groups,* […] *have the typical microconch horn developed, but show the beginnings of the macroconch type of ribbing*". Another case of male-to-female ‘sex reversal’ is reported by Brochwicz-Lewiński and Różak [19] based on a specimen of the Oxfordian Perisphinctidae *Subdiscosphinctes cracoviensis* (Siemiradzki) figured by Méléndez and Fontana [31]. This specimen displays the onset of macroconch ornamentation [i.e. assigned to the subgenus *S. (Aureimontanites)* or *Larcheria*], after a juvenile peristome typical of the microconchs. The most recent documented case is to be found within the Kimmeridgian Aspidoceratidae dimorphic pair *Physodoceras* [M]—*Sutneria* [m] [10] (Fig. 9B). These authors illustrated a specimen of *Sutneria subeumela* bearing lappets, which shows a bituberculate sculpture stage in the sub-adult whorls typical for females. In summary, the Mesozoic ammonoids with apparent ‘sex reversals, either male-to-female or female-to-male, belong to the superfamily Perisphinctoidea (families Perisphinctidae or Aspidoceratidae) and occur between the Callovian and the Kimmeridgian stages.

Many pathologies modifying the shell geometry and/or ornamentation of ammonoids are documented in the fossil record and classified into categories called forma-types (see review by Hengsbach [32]) [33]. These forma-types usually result from exogenic causes (e.g., sublethal injury, parasitism) and their expressions reflect the developmental response of the ammonoids to a perturbation. The study of such ‘monsters’ has greatly contributed to the understanding of ammonoid evolution and biology in the past decades [34] and references therein). To our knowledge, no one ever defined a forma-type for ammonoids showing apparent ‘sex reversals’ during shell growth. We, therefore, erect a new forma-type pathology here named “forma hermaphrodita” (from Hermaphroditos, the Greek god who displayed both characteristics of male and female). We include the specimens of Brochwicz-Lewiński and Róża [19], Parent et al. [10] and the *P. athleta* individuals from Méron in the “forma hermaphrodita” pathology. None of these specimens presents any clear evidence for injury or parasitism although the early whorls are not preserved for further confirmation. Note that the specimen mbe.1401 of *P. athleta* has anarchic stage (*ii*) that could pinpoint some perturbations during the early growth.

Based on the published cases listed above, all the individuals referred to as “forma hermaphrodita” belong to perisphinctoid species in which a classic sexual dimorphism is encountered. The rarity of such “forma hermaphrodita” specimens in the fossil record thus pinpoints a pathological nature for explaining these apparent ‘sex reversals’.

### ‘Sex reversals’ in the Ammonoidea and their significance

Little is known about the ammonite soft parts [8, 35], and it is usually not possible to determine which internal sexual organs occur in a specimen, which has both male and female external shell features. In gonochoric species, the anomalous case of individuals possessing gonadal tissue of one sex, but which exhibit external phenotype of the opposite sex has been previously referred to as pseudohermaphroditism. According to Lee et al. [36], pseudohermaphroditism is only a manifestation of anomalous sex development among a large mosaic of sex disorders observed in gonochoric species and the term intersexuality should be preferentially used. Considering that the Ammonoidea is a strictly gonochoristic group like modern cephalopods [3], the new “forma hermaphrodita” pathology likely illustrates intersex specimens. This conforms with a previous suggestion made by Cope [30].

The modern cephalopods group strictly gonochoric species, but external sexual features are generally lacking [37]. According to Rocha et al. [37], the sexual distinction is most often based on the presence of a hectocotylized arm in males, which transfers the spermatophores to the females. Cases of intersexuality have been reported by Ortiz and Ré [38] and Hoving et al. [39]. The abnormal cephalopod specimen of *Enteroctopus megalocyathus* described by Ortiz and Ré [38] is sexed as a female (i.e. absence of the hectocotylus) but shows internally male structures with normal genital female characteristics and orientated as in normal octopuses. The authors pointed out that ''*the presence of mixed female and male structures may not have caused sterility for the female function*" (Ortiz and Ré [38], p. 321). The intermediate-sized squid specimens of *Ancistrocheirus lesueurii* described by Hoving et al. [39] show female nidamental glands in the mantle cavity associated with a normally developed male reproductive system. According to the authors, these intersex specimens are common in the population and these ‘sex reversals’ did "*not seem to affect male functionality and is apparently advantageous in that larger body size is accompanied by larger testis and spermatophores''*.

In these cases, the causes of intersexuality are not clearly established but environmental pollutants arising from human activity are evoked. According to Hoving et al. [39], environmental pollutants may have conducted to abnormal feminisation and/or masculinisation because they act as endocrine disrupters. ‘Sex reversals’ and/or non-functional reproductive abnormalities have also been documented in various gonochoric gastropods and linked to endocrine disrupters (Oberdorster and Cheek [40]). Sexual pathologies in gastropods can also result from the infestation by trematod larvae, which stimulate or inhibit neural-endocrinal activity by direct gonadal influence [41]. This infestation ultimately leads to feminisation or masculinisation. Besides, genetic abnormalities [42], temperature fluctuations [43] or viruses [44] are mentioned in intersex fishes, isopods and crustaceans as causes for intersexuality.

The Middle to Upper Jurassic transition is not recognised as a critical period for the biosphere, which had to face deep climatic disturbances, except maybe in the Boreal Realm [45]. This period records a low magnitude, short-term global climate cooling at the Upper Callovian–Middle Oxfordian boundary followed by a subsequent warming in the Upper Oxfordian–Lower Kimmeridgian [46]. At that time, the perisphinctoid ammonites became extremely diverse and have colonised a wide range of habitats in different bioprovinces of the world [47]. Thus, it can hardly be argued that intersexuality in the Perisphinctoidea point to the occurrence of a new reproductive strategy illustrating a veritable hermaphroditism due to changing environmental conditions. Regardless of whether “forma hermaphrodita” is due to an exogenic (infestation, virus) or endogenic cause (genetic abnormalities), the high frequency of intersex Perisphinctoidea in the Jurassic deposits can be explained by the two observations: (1) the easy recognition of dimorphic pairs, and (2) the abundance of more or less adult and sufficiently preserved fossil palaeopopulations in which intersex specimens have statistically higher chances to be found (see also discussion in Klug et al. [3], §7.3.7.3).

## Conclusions

We here document a new case of 'sex reversal' in ammonoid shells, based on two specimens of the Callovian Aspidoceratidae *Peltoceras athleta*. Those specimens have started with a female ontogeny and show an apparent change toward maleness in the adult. Other cases of female-to-male ‘sex reversal’, as well as male-to-female ‘sex reversals’ are known from the ammonoid record, all belonging to the Jurassic superfamily Perisphinctoidea (families Perisphinctidae or Aspidoceratidae). These ‘sex reversals’ are pathological in nature and are herein referred to the new forma-type pathology “forma hermaphrodita”. Regardless of whether the “forma hermaphrodita” is due to an exogenic or endogenic cause, those specimens illustrate pathologic cases of intersexuality in the Ammonoidea.

## Data Availability

The Méron specimens are deposited in the private collection of P-YB under collection number mbe.9305 and mbe.1404. Arrangements are taken in his will to leave these specimens in a public collection. Plastic casts are also housed in the Frau collection—with same collection numbers—that is being deposited at the Natural History Museum of Aix-en-Provence, France.

## Abbreviations

D: Maximum diameter
Uw: Umbilical width
Wh: Whorl height
Ww: Whorl width
K: Number of ribs per half a whorl
[m]: Microconchs
[M]: Macroconchs
